# Supplementary material for: Accelerating ultrafast magnetization reversal by non-local spin transfer
Source: Nat Commun. 2023 Jan 27;14:445. doi: 10.1038/s41467-023-36164-1 (PMC9883451; doi:10.1038/s41467-023-36164-1)
Supplement: Supplementary file 1 — Supplementary Information [file 41467_2023_36164_MOESM1_ESM.pdf]

Accelerating ultrafast magnetization reversal by non-local spin transfer

Quentin Remy[†], Julius Hohlfeld[†], Maxime Vergès[†], Yann LeGuen[†], Jon Gorchon[†], Grégory Malinowski[†], Stéphane Mangin[†], Michel Hehn[†]

[†] Université de Lorraine, Institut Jean Lamour, UMR CNRS, Nancy 7198, France

Supplementary Figures

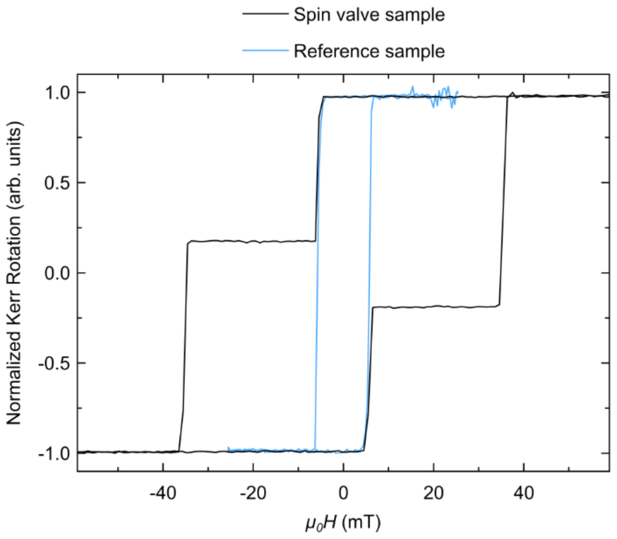

Supplementary Fig.1 | Hysteresis loop of spinvalve and reference samples. Normalized Kerr rotation as a function of the applied external magnetic field for the reference (blue) and the spin valve (black) samples.

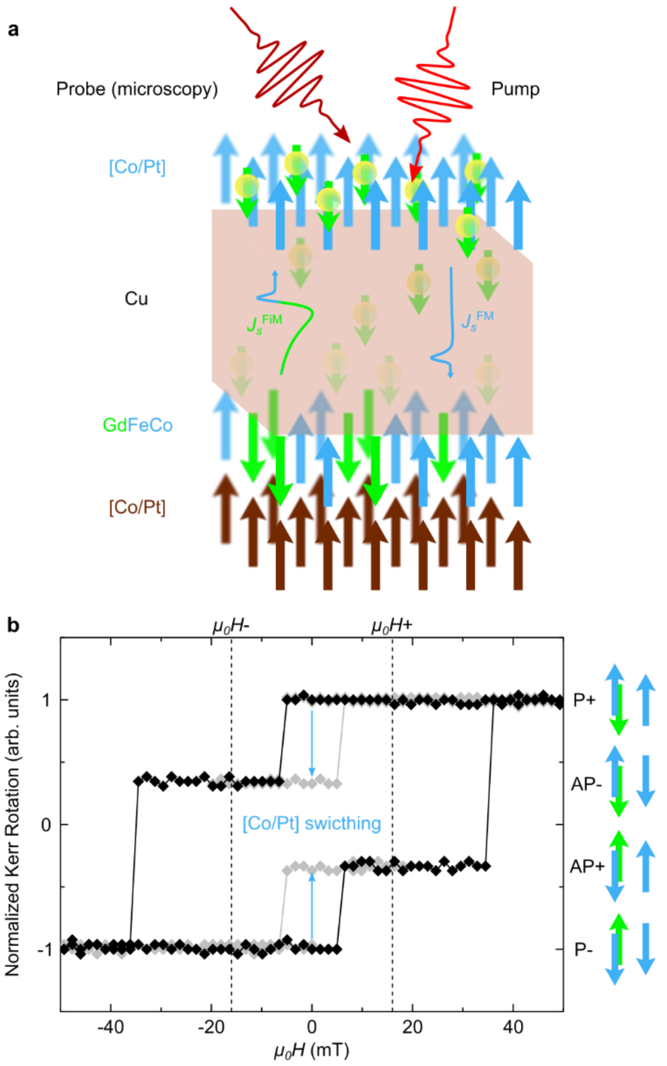

Supplementary Fig.2 | Experimental design to generate and probe the ultrafast magnetization reversal of a ferromagnetic layer. a, Scheme of spin valve where blue and green arrows represent the transition metal and Gd magnetization, respectively. The same color code is used to indicate the origin of spin accumulation (arrows with bullets) and the temporal evolution and propagation direction (indicated by the arrow on the curved line) of spin currents J_s^{FiM} and J_s^{FM} which is given by the curved lines. A blue color represents a spin current polarization with the same direction as the transition metal magnetization while a green color represents a polarization with the same direction as the Gd magnetization. The brown arrows represent the extra [Co/Pt] multilayer. **b**, Typical major (black) and minor (gray) hysteresis loops measured via the Kerr rotation, measured on our TR-MOKE microscope, as function of a perpendicular external field, when the pump laser is blocked. There is no exchange coupling between the soft free- and hard pinned-layer and the four stable remanent states observed at zero field correspond to the labeled magnetization configurations indicated by the arrows. The same conventions as in panel a) are assumed for the representation of magnetization. The dashed lines below μ_0H+ and μ_0H- indicate the value of the applied external magnetic field for TR-MOKE microscopy measurements in + (P+ and AP+) and - (P- and AP-) configurations. The thin blue arrows in the minor hysteresis loops indicate the change of MOKE signal upon reversal of the (top) single [Co/Pt] multilayer.

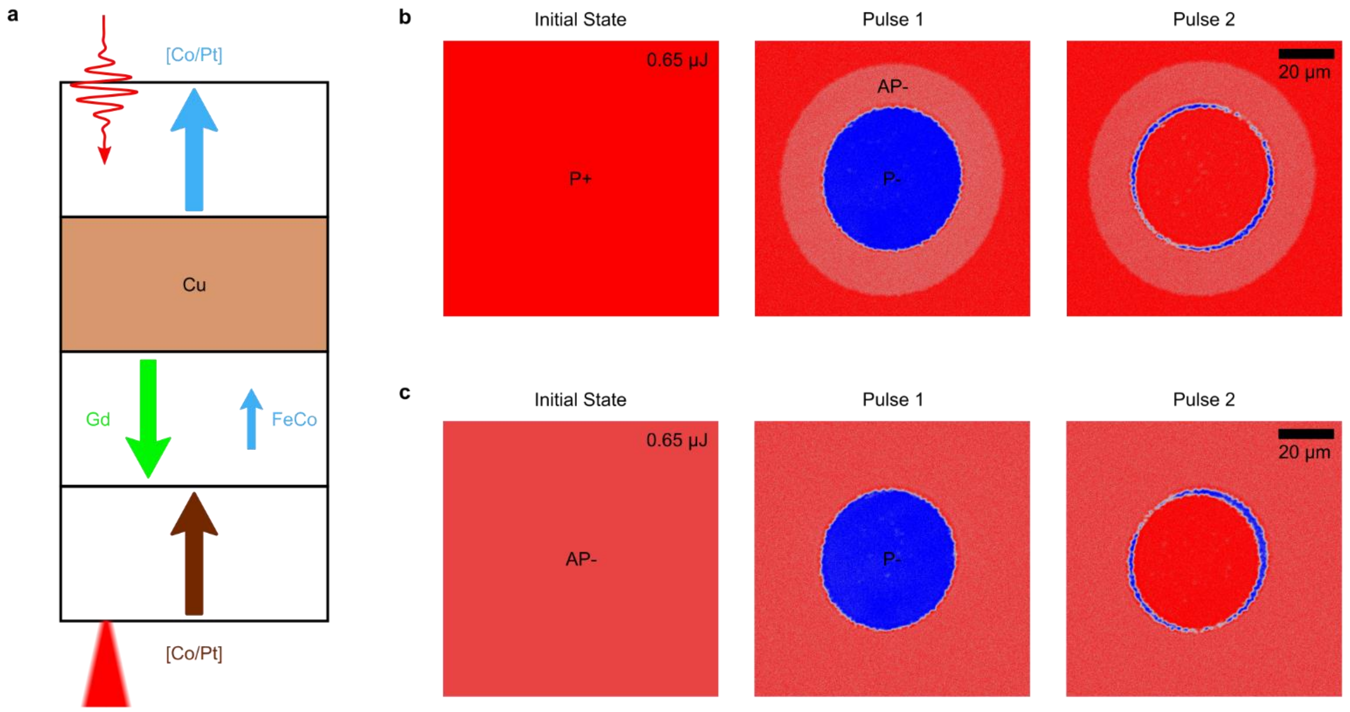

Supplementary Fig.3 | Static MOKE images after single laser pulse irradiation. a, Sample and the experimental configuration. The laser pulse irradiates the top side of the sample in standard (static) MOKE microscopy (shown as the red cone) is performed from the ferrimagnetic layer (through the substrate) side. The same color code as in Figure 2 is used. **b**, **c** Results of sending one and two laser pulses on the sample which has either been saturated in the P+ configuration (**b**) or the AP+ configuration (**c**) beforehand. The pulse energy is 0.65 µJ in all cases. The central area exhibits helicity independent all optical (toggle) switching because of the GdFeCo alloy.

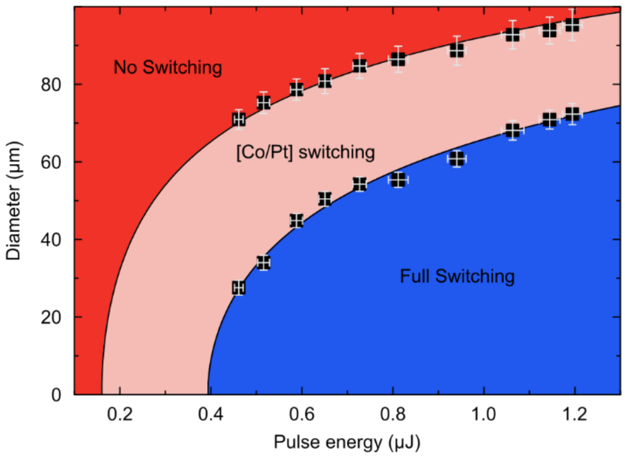

Supplementary Fig.4 | Magnetic domain diameter as a function of the incident laser pulse energy. The solid black lines indicate the (global) fit obtained from measurements with opposite saturation magnetization. For GdFeCo (full switching), the fit was also performed for data obtained from both parallel and antiparallel configurations. These fits allow us to extract the threshold fluence for the different magnetic reversals.

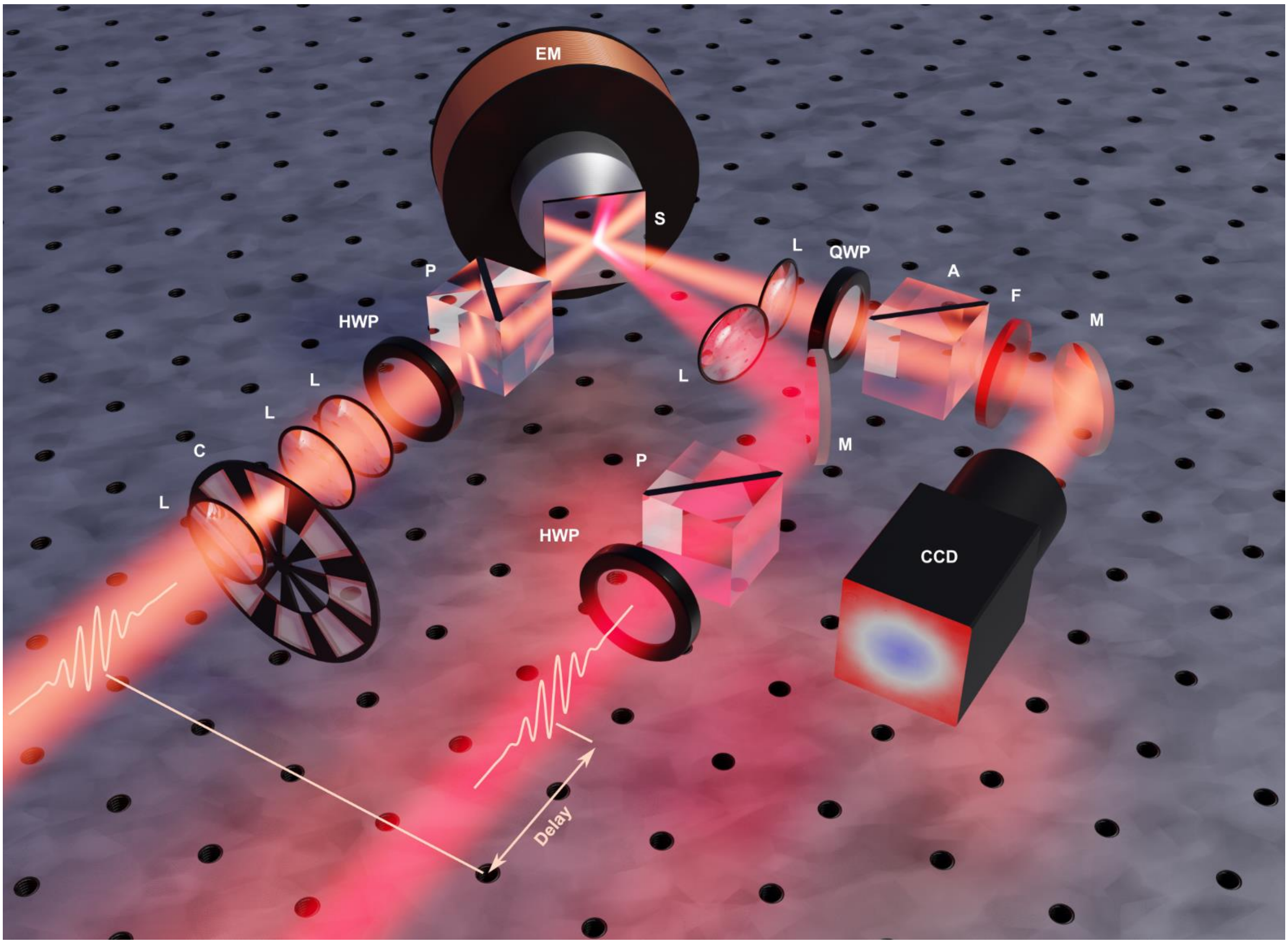

Supplementary Fig.5 | Time resolved magneto-optical Kerr effect microscopy setup. The TR-MOKE microscope contains the following elements: half wave-plate (HWP); quarter wave-plate (QWP); polarizer (P); analyzer (A); chopper (C); sample (S); electromagnet (EM); mirror (M); lens (L); filter (F); charge coupled device (CCD). The pump (800 nm; red) and probe (850 nm; orange for the purpose of illustration) beam angles of incidence are realistic. A delay line (not shown) is used to control the pump-probe delay.

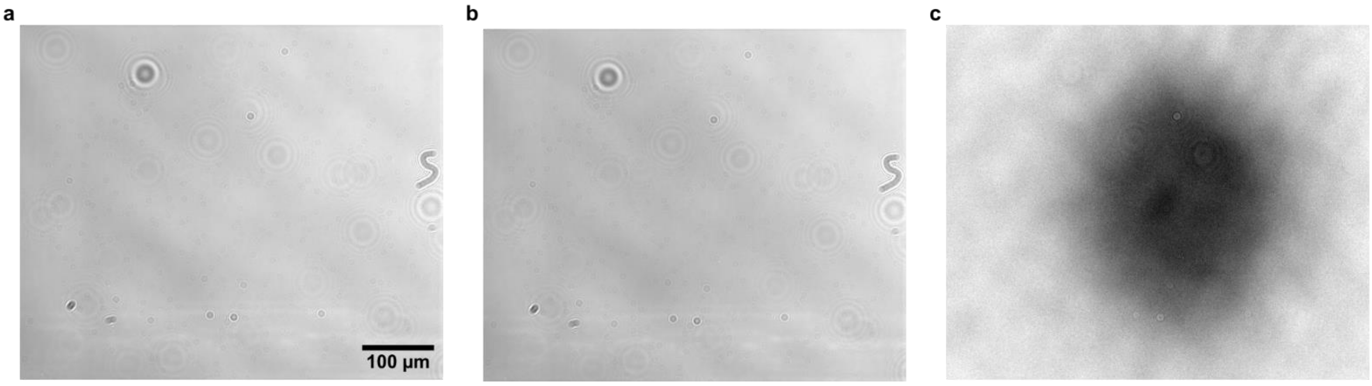

Supplementary Fig.6 | Background subtraction of microscopy images. **a**, MOKE microscopy image at negative delay where there is no signal. **b**, MOKE microscopy image at a pump-probe delay of 1 ps. **c**, Result of subtracting the background image **a** to the image at positive delay **b**. The example of a P- configuration is shown. The raw images in figures **a** and **b** display the full range of grey values while the scale has been changed in **c** to make the signal more visible. This signal needs to be analysed in order to retrieve the magnetization of each magnetic layer, as explained in the Method section and the Supplementary Information.

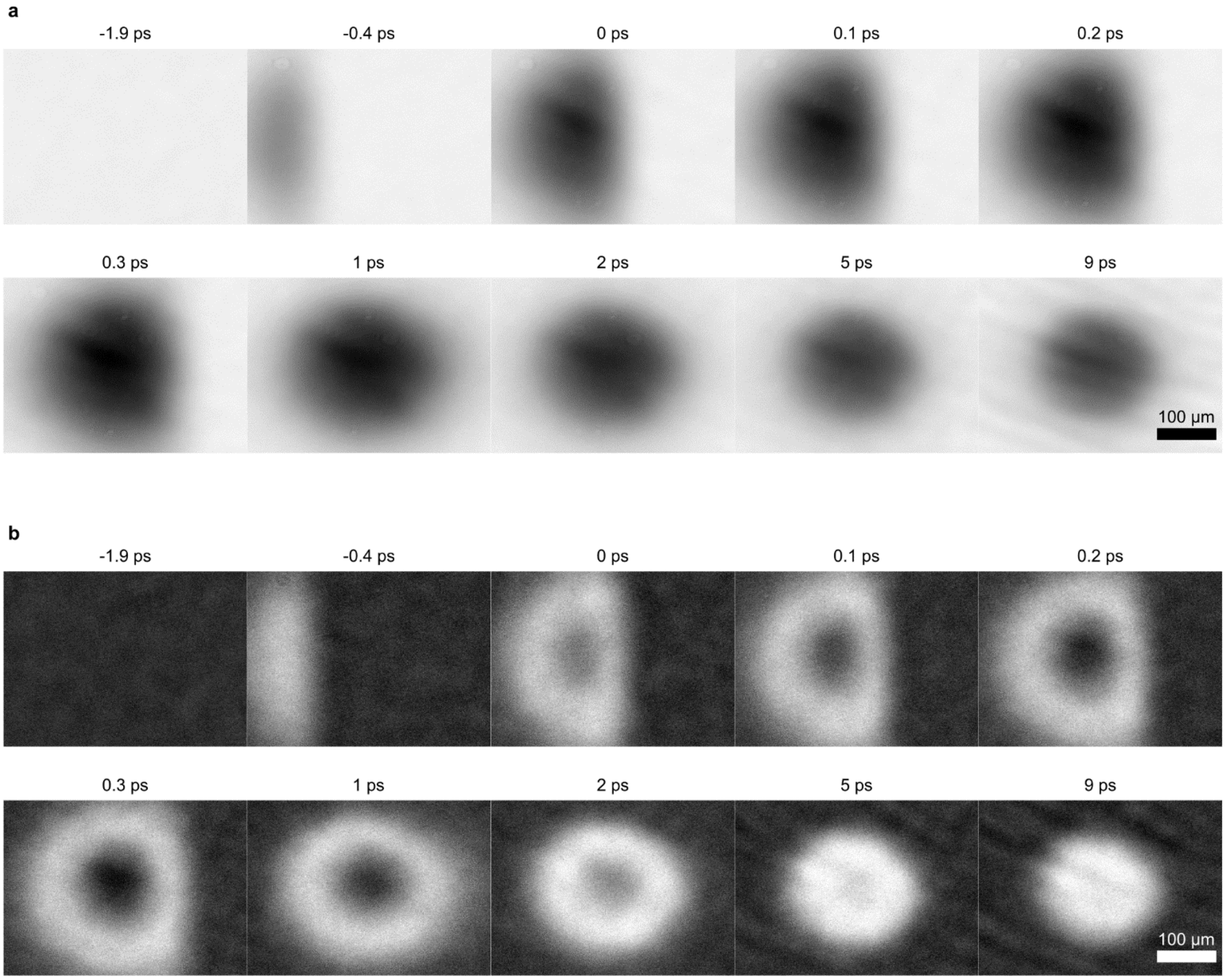

Supplementary Fig.7 | TR-MOKE microscopy data after background subtraction for various pump-probe delays. a, **b**, Time dependent MOKE images (raw data with background at negative pump-probe delay subtracted and the images are rescaled horizontally to correct for the probe angle of incidence), obtained with the TR-MOKE microscopy setup, of the spin valve sample for a laser power of 120 mW and various pump-probe delays for an initial magnetic configuration which is either P (**a**) or AP (**b**) and a given quarter wave-plate setting. The image contrast was adjusted such that one can clearly see the magnetization pattern in each case. In (b), the color code is such that a brighter contrast corresponds to a signal dominated by [Co/Pt] while a darker one corresponds to a signal dominated by GdFeCo.

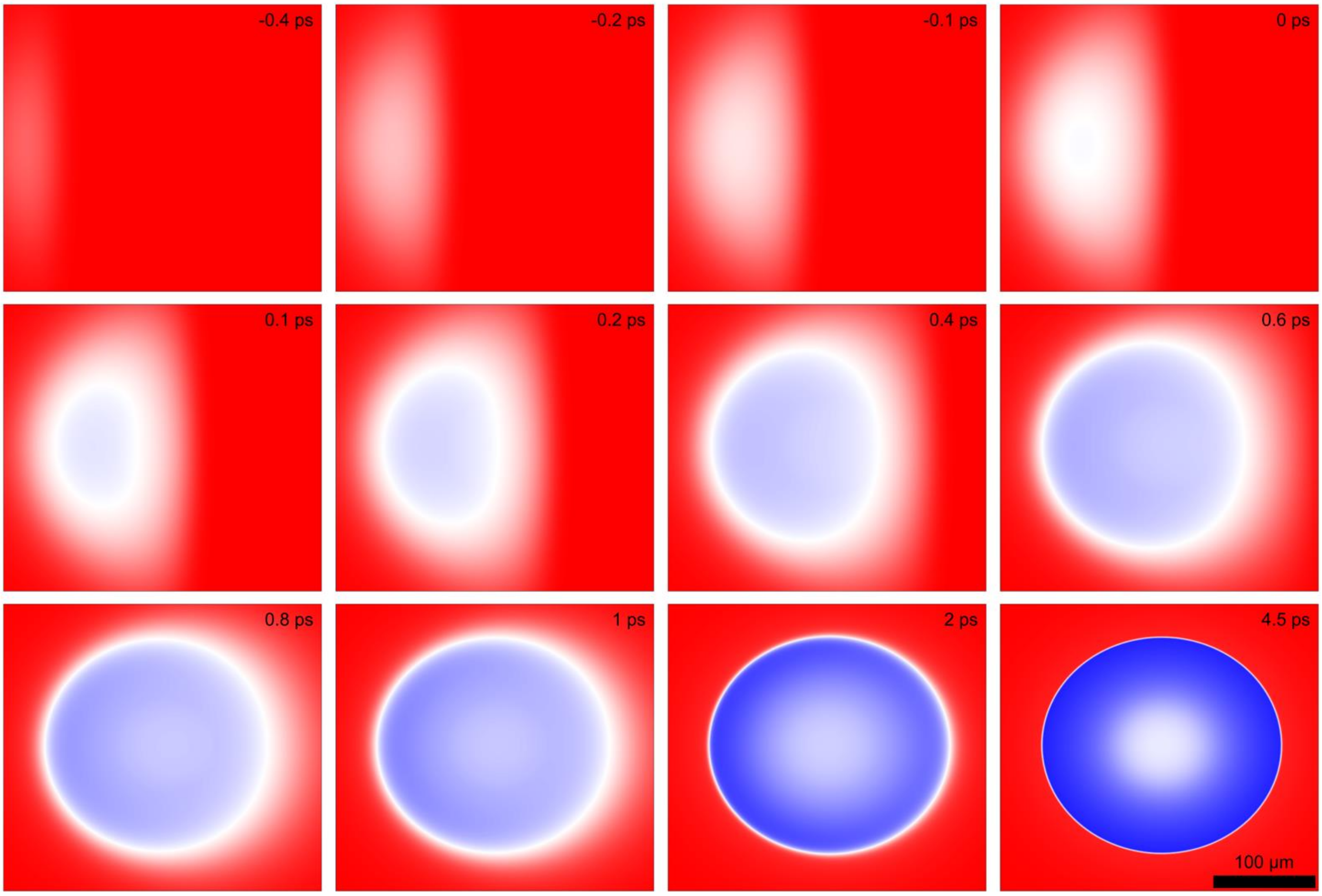

Supplementary Fig.8 | TR-MOKE microscopy simulations. Simulations of the TR-MOKE microscopy images, if only the magnetization of the [Co/Pt] multilayer was probed, for different pump-probe delays (defined as zero when the peak of the laser pulse reaches the center of the image) for a laser power of 120 mW and the other experimental parameters as detailed in the method section. The simulations do not consider the horizontal scaling of the images due to the probe angle as it is corrected in the experimental data shown in Supplementary Fig. 7. The color code is the same as for MOKE microscopy images.

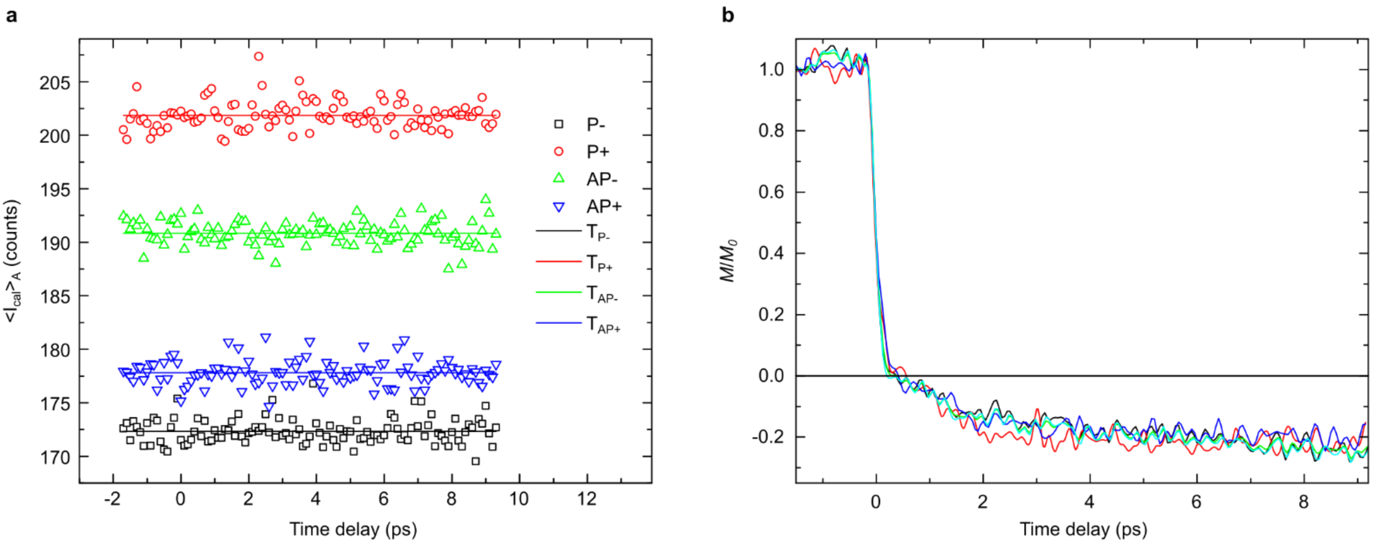

Supplementary Fig.9 | Experimental fluctuations. a, Averaged intensity $<I_{cal;i}(\tau)>_A$ within a predefined area A as a function of time delay for all magnetic configurations i in {P+, P-, AP+, AP-}. The straight lines show the target values i.e. the average over time delay $T_i = <<I_{cal;i}(\tau)>_A>$. **b**, Individual magnetization dynamics resulting from the analysis with five different pairs of quarter wave-plate angles, shown in different colors, for a laser fluence of 5.08mJ/cm^2. The error bars shown in this work are the standard deviations obtained from the averaging of such curves.

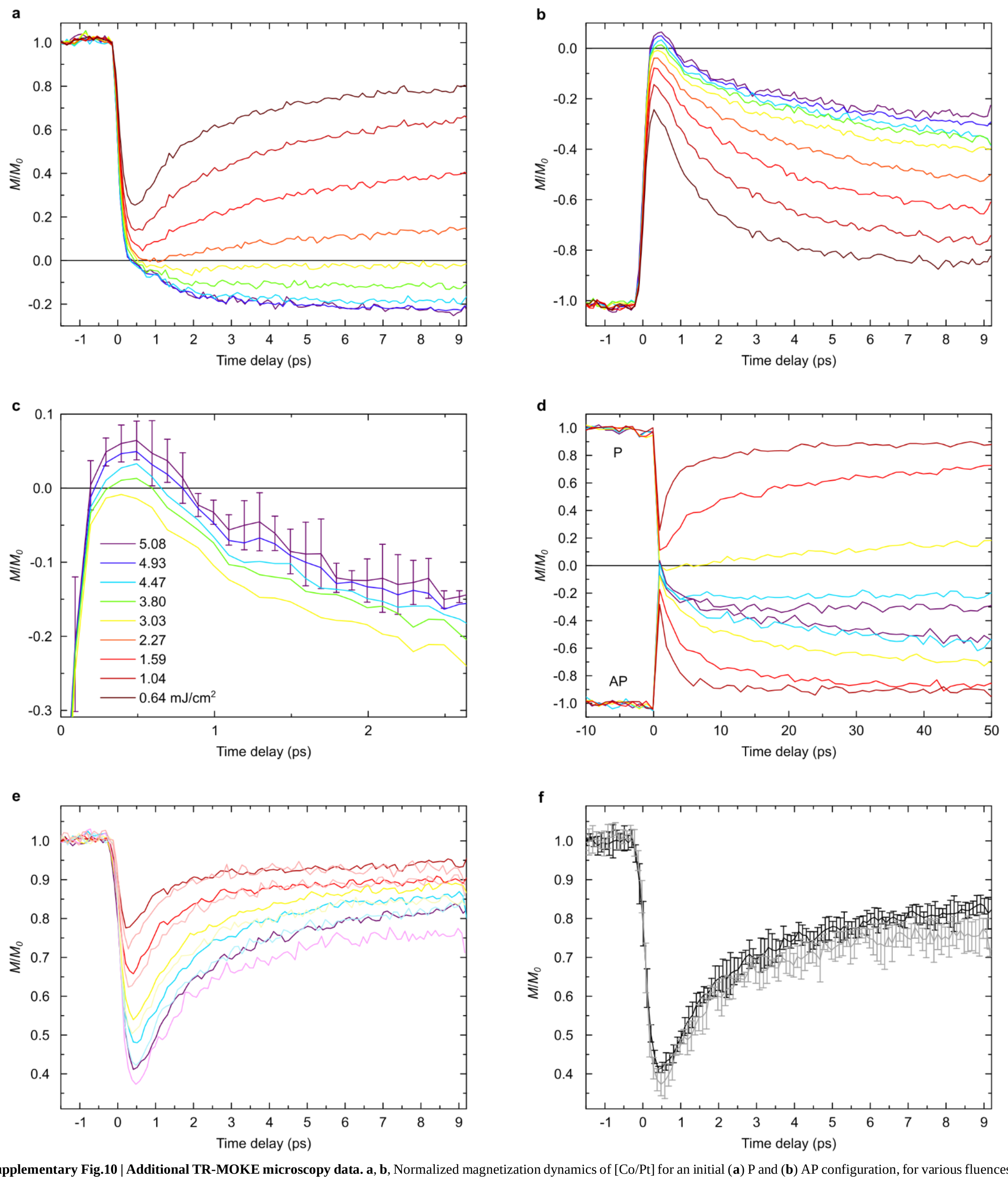

Supplementary Fig.10 | Additional TR-MOKE microscopy data. a, **b**, Normalized magnetization dynamics of [Co/Pt] for an initial (**a**) P and (**b**) AP configuration, for various fluences. **c**, Zoom on the region around the magnetization overshoot where error bars are shown. **d**, Same data as in Fig.3a but for longer time delays. **e**, Normalized magnetization dynamics of GdFeCo for both initial P (saturated colors) and AP (light colors) configurations, as a function of pump-probe delay. **f**, Normalized magnetization dynamics of GdFeCo for 5.08 mJ/cm^2 and both initial magnetic configurations (P in black and AP in grey) with error bars. The color code for fluences is indicated in **c**.

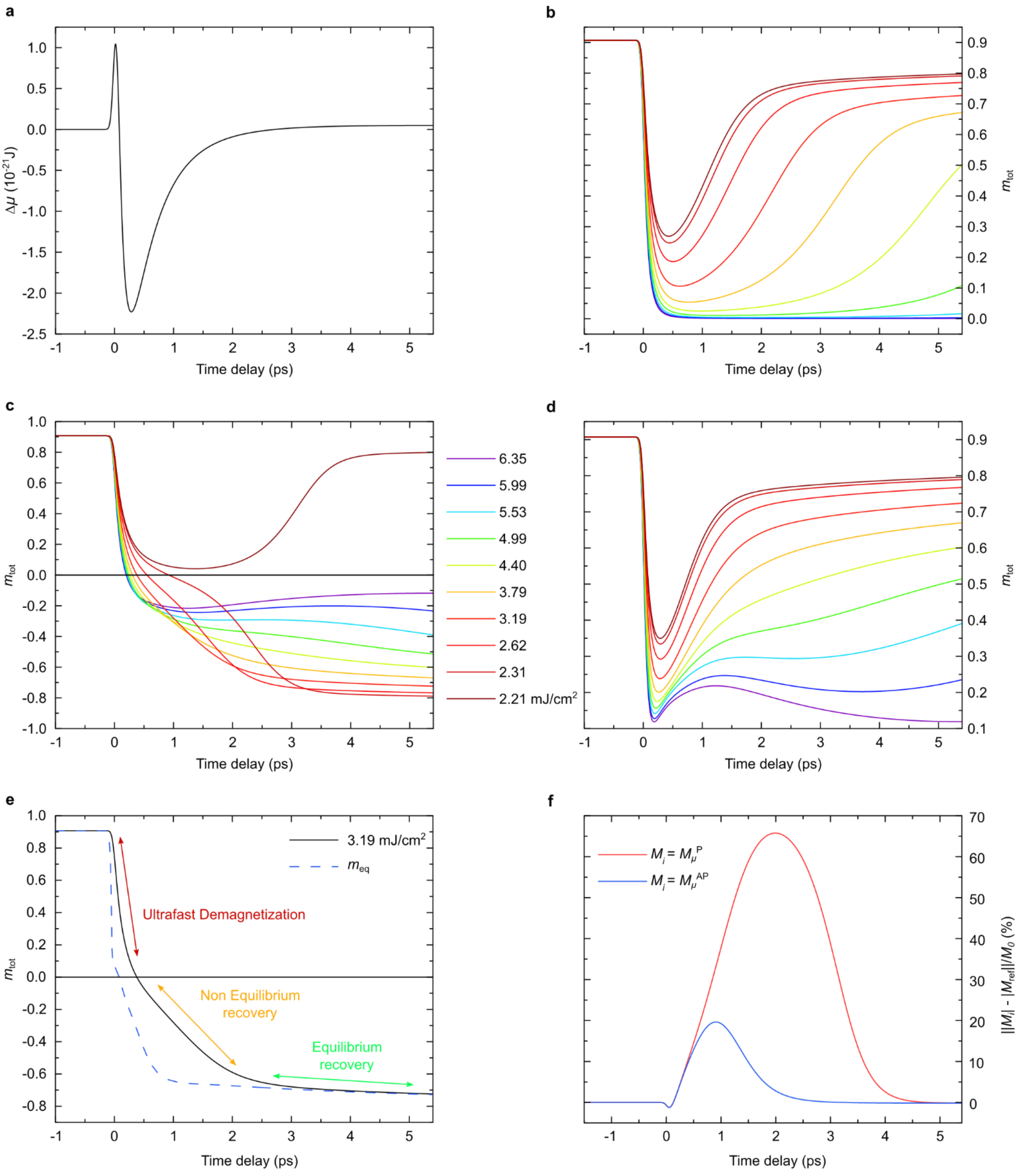

Supplementary Fig.11 | Additional simulations of the ultrafast magnetization dynamics of the [Co/Pt] ferromagnetic multilayer. **a**, Predicted spin accumulation in the [Co/Pt] multilayer as a function of time delay for a fluence of 6.64 mJ/cm^2 for an AP configuration. **b**, **c**, **d**, Simulations of the [Co/Pt] multilayer magnetization dynamics as a function of time delay for various fluences in the spinvalve sample if there was no spin accumulation (**b**) and if there is a spin accumulation as in the P (**c**) and AP (**d**) initial configurations. **e**, Predicted total normalized magnetization dynamics of the [Co/Pt] multilayer in the P configuration for a fluence of 3.19 mJ/cm^2, and its time dependent equilibrium value, as a function of time. The time dependent equilibrium magnetization m_{eq}, towards which m(t) would tend to within an infinite time at constant electronic temperature $T_e(t)$ and spin accumulation $\Delta\mu(t)$, given by the 2TM and Supplementary Fig.11a respectively, at time t. One can see that the three steps of the reversal dynamics (Ultrafast Demagnetization, Non Equilibrium recovery and Equilibrium recovery) as observed in Fig.1 are reproduced. **f**, Predicted spin heating and spin cooling, as defined in the main part of the text, calculated from the simulation of **b**, **c** and **d** for 2.21 mJ/cm^2. This is the highest simulated fluence that does not allow a switching in the P configuration and thus where spin heating is expected to be the highest.

Supplementary information

Supplementary Methods

Measurement of magnetization via MOKE

The electric field vector amplitude of the beam transmitted to the photodetector E^t is related to the s-polarized electric field incident on the sample E^i via:

$$\begin{bmatrix} E_p^t \\ E_s^t \end{bmatrix} = \mathbf{AQS} \begin{bmatrix} 0 \\ E_s^i \end{bmatrix} = \begin{bmatrix} \cos^2(\alpha) \cdot r'_{ps} + \sin(\alpha)\cos(\alpha) \cdot r'_{ss} \\ \sin^2(\alpha) \cdot r'_{ss} + \sin(\alpha)\cos(\alpha) \cdot r'_{ps} \end{bmatrix} E_s^i \qquad (S1)$$

where the following Jones matrices of the analyzer **A,** the quarter wave plate **Q,** and of the sample **S** are used:

$$\mathbf{A} = \begin{bmatrix} \cos^2(\alpha) & \sin(\alpha)\cos(\alpha) \\ \sin(\alpha)\cos(\alpha) & \sin^2(\alpha) \end{bmatrix} \qquad (S2)$$

$$\mathbf{Q} = \begin{bmatrix} \cos^2(\theta) + i\sin^2(\theta) & (1-i)\sin(\theta)\cos(\theta) \\ (1-i)\sin(\theta)\cos(\theta) & \sin^2(\theta) + i\cos^2(\theta) \end{bmatrix} \qquad (S3)$$

$$\mathbf{S} = \begin{bmatrix} r_{pp} & r_{ps} \\ r_{sp} & r_{ss} \end{bmatrix} \qquad (S4)$$

The diagonal reflection coefficients $r_{pp} = r_{pp,0}\exp(i\delta_{pp})$ and $r_{ss} = r_{ss,0}\exp(i\delta_{ss})$ are to first order independent of the sample magnetization[1]. The off-diagonal coefficients scale linearly with the normalized magnetization m, because of the small Kerr angles and ellipticities, and will be expressed as:

$$r_{ps}(m) = -r_{ps}(-m) = -r_{sp}(m) = m \cdot r_{ps,0}\exp\left(i\delta_{ps}\right) \qquad (S5)$$

The reflection coefficients r'_{ss} and r'_{ps} are the reflection coefficients of the sample modified by the quarter wave plate and are given by:

$$\begin{bmatrix} r'_{ss} \\ r'_{ps} \end{bmatrix} = \exp\left(-\frac{i\pi}{4}\right) \cdot \begin{bmatrix} (1-i)\sin(\theta)\cos(\theta) \cdot r_{ss} + (\cos^2(\theta) + i\sin^2(\theta)) \cdot r_{ps} \\ (\sin^2(\theta) + i\cos^2(\theta)) \cdot r_{ss} + (1-i)\sin(\theta)\cos(\theta) \cdot r_{ps} \end{bmatrix} \qquad (S6)$$

The intensity measured at the detector, normalized by the intensity of the incoming beam is given by:

$$\frac{I^t}{I^i} = \frac{r_{ss,0}^2}{2}[\sin^2(\alpha) + \sin^2(\alpha - 2\theta)] + \frac{m^2 r_{ps,0}^2}{2}[\cos^2(\alpha) + \cos^2(\alpha - 2\theta)] + \frac{r_{ss,0}^2}{2}\{\theta_K^s[\sin(2\alpha) - \sin(2\alpha - 4\theta)] + \eta_K^s\, 2\sin(2\alpha - 2\theta)\} \quad (S7)$$

where the definition of the complex Kerr angle:

$$\Theta_K^s = \frac{r_{ps}}{r_{ss}} = \theta_K^s + i\eta_K^s = m \cdot \left(\theta_{K,0}^s + i\eta_{K,0}^s\right) \quad (S8)$$

is used. Here, θ_K^s and η_K^s represent the Kerr rotation angle and Kerr ellipticity, which both scale linearly with m. Obviously, signals measured for one particular orientation of the magnetization might have significant contributions that are either non-magnetic (first term in equation (S7)) or scale as m^2 (second term in equation (S7)). This obstacle can be overcome by subtracting the signals measured for opposite magnetizations:

$$\Delta I_m^t = \frac{I^t(m) - I^t(-m)}{I^i} = m \cdot r_{ss,0}^2\left\{\theta_{K,0}^s[\sin(2\alpha) - \sin(2\alpha - 4\theta)] + \eta_{K,0}^s\, 2\sin(2\alpha - 2\theta)\right\} = m \cdot 2r_{ss,0}^2\left\{\theta_{K,0}^s \cos(2\alpha - 2\theta)\sin(2\theta) + \eta_{K,0}^s \sin(2\alpha - 2\theta)\right\} \quad (S9)$$

Note, that the angle of the QWP changes the weight of the contributions from the Kerr rotation θ_K^s and Kerr ellipticity η_K^s to the measured signal. In particular the settings $\theta = \alpha$ and $\theta = 0$ lead to results that depend solely on θ_K^s and η_K^s, respectively:

$$\Delta I_m^t(\theta = \alpha) = m \cdot 2r_{ss,0}^2 \sin(2\alpha)\, \theta_{K,0}^s \quad (S10)$$

$$\Delta I_m^t(\theta = 0) = m \cdot 2r_{ss,0}^2 \sin(2\alpha)\, \eta_{K,0}^s \quad (S11)$$

The angle $\theta = \alpha$ leads to signals that are identical to the ones measured without QWP as expected.

Determination of the magnetization as a function of pump-probe delay for different layers

All the considerations above are valid for magnetic multilayers with an arbitrary number of layers and layer thicknesses. For a multilayer system in the thin film limit[1], which is fullfilled for our spinvalve, the total complex Kerr angle $\Theta_K^{s,\mathrm{tot}}$ is the sum of the complex Kerr angles of individual layers $\Theta_K^{s,i}$:

$$\Theta_K^{s,\mathrm{tot}} = \frac{r_{ps}^{\mathrm{tot}}}{r_{ss}^{\mathrm{tot}}} = \sum_{i=1}^{n} \Theta_K^{s,i} = \sum_{i=1}^{n} \theta_K^{s,i} + i \sum_{i=1}^{n} \eta_K^{s,i} = \theta_K^{s,\mathrm{tot}} + i\eta_K^{s,\mathrm{tot}} \qquad (S12)$$

where the sum runs over all n magnetic layers. Note, that the individual values of $\Theta_K^{s,i}$ are not just dependent on the material properties of the i-th layer but also on its position within the stack and the optical properties and thicknesses of all layers. Since measurements for different QWP angles θ lead to different sensitivity to $\theta_K^{s,\mathrm{tot}}$ and $\eta_K^{s,\mathrm{tot}}$, they will simultaneously have different sensitivity to the magnetization of individual layers if the phase of the complex Kerr angle of those layers is different, i.e. if $\theta_K^{s,i}/\eta_K^{s,i} \neq \theta_K^{s,j}/\eta_K^{s,j}$ for $i \neq j$. For the signals measured as a function of pump-probe delay τ in the P and AP configurations in our collinear spinvalve for different QWP settings i it follows:

$$p_i(\tau) = \frac{\Delta I_{\mathrm{P}}^t(\tau)}{\Delta I_{\mathrm{P}}^t(\tau < 0)} = x_i(\tau) \cdot m_{\mathrm{pinned}}(\tau) + y_i(\tau) \cdot m_{\mathrm{free}}(\tau) \qquad (S13)$$

$$ap_i(\tau) = \frac{\Delta I_{\mathrm{AP}}^t(\tau)}{\Delta I_{\mathrm{AP}}^t(\tau < 0)} = x_i(\tau) \cdot m_{\mathrm{pinned}}(\tau) - y_i(\tau) \cdot m_{\mathrm{free}}(\tau) \qquad (S14)$$

Here, m denotes values of the magnetization normalized such that the normalized magnetization of the free m_{free} and pinned m_{pinned} layers satisfy $m_{\mathrm{free}}(\tau < 0) = m_{\mathrm{pinned}}(\tau < 0) = 1$, and the weight factors for the magnetization of the individual layers are given by:

$$x_i(\tau) = \frac{f_{\mathrm{pinned},i}(\tau)}{f_{\mathrm{pinned},i}(\tau < 0) + f_{\mathrm{free},i}(\tau < 0)} \qquad (S15)$$

$$y_i(\tau) = \frac{f_{\mathrm{free},i}(\tau)}{f_{\mathrm{pinned},i}(\tau < 0) + f_{\mathrm{free},i}(\tau < 0)} \qquad (S16)$$

with:

$$f_{\mathrm{L},i}(\tau) = 2\left[r_{ss,0}^{\mathrm{tot}}(\tau)\right]^2 \left\{\theta_{K,0,\mathrm{L}}^{s}(\tau)\cos(2\alpha - 2\theta_i)\sin(2\theta_i) + \eta_{K,0,\mathrm{L}}^{s}(\tau)\sin(2\alpha - 2\theta_i)\right\} \tag{S17}$$

and L $\in$ {pinned, free}. Note, that at our probe wavelength of 850 nm the magneto-optical Kerr effect probes only the Fe and Co content of the pinned layer[2]. Consequently, m_{pinned} corresponds to the transition metal magnetization and not the total magnetization of the pinned layer. For a given magnetic configuration of the spin valve S, the magnetization dynamics in the free and the pinned layers can be derived from the corresponding transients for two different QWP settings i and j via:

$$\begin{pmatrix} m_{\mathrm{free}}(\mathrm{S},\tau) \\ m_{\mathrm{pinned}}(\mathrm{S},\tau) \end{pmatrix} = \frac{1}{y_i(\tau)x_j(\tau) - y_j(\tau)x_i(\tau)} \begin{pmatrix} x_j(\tau) & -x_i(\tau) \\ -y_j(\tau) & y_i(\tau) \end{pmatrix} \begin{pmatrix} s_i(\tau) \\ s_j(\tau) \end{pmatrix} \tag{S18}$$

with $s = p(\tau)$ for S = P and $s = ap(\tau)$ for S = AP. However, this derivation would require knowledge of the time dependencies of the weight factors x and y which cannot be determined experimentally without knowledge of $m_{\mathrm{pinned}}(\tau)$ and $m_{\mathrm{free}}(\tau)$. Only the equilibrium values of the relative weights of the pinned and free layer magnetization, $x_{i,0}$ and $y_{i,0}$, can be derived from experimental data at negative delay (where by definition $m_{\mathrm{free}}(\tau < 0) = m_{\mathrm{pinned}}(\tau < 0) = 1$) via:

$$x_{i,0} = \frac{p_i(\tau < 0) + ap_i(\tau < 0)}{2} \tag{S19}$$

$$y_{i,0} = \frac{p_i(\tau < 0) - ap_i(\tau < 0)}{2} \tag{S20}$$

The standard way to solve the dilemma that the time dependencies of the weight factors and of the magnetization cannot simultaneously be derived from experimental data is to assume that changes of optical properties that are not directly related to the magnetization are negligible, i.e. that the weights are constant $x_i(\tau) = x_{i,0}$ and $y_i(\tau) = y_{i,0}$. This assumption is usually motivated by the fact that the pump and probe wavelenghts are different and that transient reflectivity changes can be neglected. Here we show that simultaneous analyses of data measured in the P and AP configurations of the spin valve for at least three different angles of the QWP can be used to validate this assumption. Analyzing data measured for two

different angles θ_i and θ_j of the QWP under the assumption of $x_i(\tau) = x_{i,0}$ and $y_i(\tau) = y_{i,0}$ in terms of equation (S18) yields:

$$m_{\text{free,theo}}(\tau) = \frac{f_{\text{pinned},j,0} f_{\text{pinned},i}(\tau) - f_{\text{pinned},i,0} f_{\text{pinned},j}(\tau)}{f_{\text{pinned},j,0} f_{\text{free},i,0} - f_{\text{pinned},i,0} f_{\text{free},j,0}} \cdot m_{\text{pinned,exp}}(\tau) + \frac{f_{\text{pinned},j,0} f_{\text{free},i}(\tau) - f_{\text{pinned},i,0} f_{\text{free},j}(\tau)}{f_{\text{pinned},j,0} f_{\text{free},i,0} - f_{\text{pinned},i,0} f_{\text{free},j,0}} \cdot m_{\text{free,exp}}(\tau) \qquad (S21)$$

$$m_{\text{pinned,theo}}(\tau) = \frac{f_{\text{pinned},j}(\tau) f_{\text{free},i,0} - f_{\text{pinned},i}(\tau) f_{\text{free},j,0}}{f_{\text{pinned},j,0} f_{\text{free},i,0} - f_{\text{pinned},i,0} f_{\text{free},j,0}} \cdot m_{\text{pinned,exp}}(\tau) + \frac{f_{\text{free},i,0} f_{\text{free},j}(\tau) - f_{\text{free},j,0} f_{\text{free},i}(\tau)}{f_{\text{pinned},j,0} f_{\text{free},i,0} - f_{\text{pinned},i,0} f_{\text{free},j,0}} \cdot m_{\text{free,exp}}(\tau) \qquad (S22)$$

Where "theo" refers to the normalized magnetization obtained after the analysis while "exp" refers to the real normalized magnetization. We see that non-negligible deviations of $f_{\text{L},i}(\tau)$ from $f_{\text{L},i,0}$ will in general lead to derived transients $m_{\text{free,theo}}$ and $m_{\text{pinned,theo}}$ that not only differ from $m_{\text{free,exp}}$ and $m_{\text{pinned,exp}}$, but, because i) different materials are expected to have different optical responses and ii) $\theta^s_{K,0}$ and $\eta^s_{K,0}$ would exhibit different dynamics in general[3-5], also from each other when different combinations of at least three QWP angles, e.g. i and j, j and k, and i and k, are used for their derivation. Thus by demonstrating that analyses of our data for different QWP angle combinations yield identical results for $m_{\text{free}}(\tau)$ and $m_{\text{pinned}}(\tau)$ as shown in Supplementary Fig. 9b we prove that the magneto-optical constants $\theta^s_{K,0}$ and $\eta^s_{K,0}$ of both magnetic layers remain constant. The only way in which $f_{\text{L},i}(\tau)$ might vary from $f_{\text{L},i,0}$ and still yield identical results for different QWP angle combinations is then through changes of the linear reflectivity $r^{\text{tot}}_{ss,0}(\tau)$. In this case the analysis yields:

$$m_{\text{free,theo}}(\tau) = \frac{\left[r^{\text{tot}}_{ss,0}(\tau)\right]^2}{\left[r^{\text{tot}}_{ss,0}(\tau < 0)\right]^2} m_{\text{free,exp}}(\tau) \qquad (S23)$$

$$m_{\text{pinned,theo}}(\tau) = \frac{\left[r^{\text{tot}}_{ss,0}(\tau)\right]^2}{\left[r^{\text{tot}}_{ss,0}(\tau < 0)\right]^2} m_{\text{pinned,exp}}(\tau) \qquad (S24)$$

To quantify the impact of reflectivity changes on our data we analyze the sum of signals measured in opposite magnetization configurations:

$$\frac{I^t_{\mathrm{AP}+}(\tau)+I^t_{\mathrm{AP}-}(\tau)}{I^i(\tau)}=\left[r^{\mathrm{tot}}_{ss,0}(\tau)\right]^2[\sin^2(\alpha)+\sin^2(\alpha-2\theta)]$$
$$+\left[r^{\mathrm{tot}}_{ss,0}(\tau)\right]^2[\cos^2(\alpha)+\cos^2(\alpha-2\theta)]\cdot\left\{\begin{array}{c}\left[m_{\mathrm{free}}(\tau)\theta^s_{K,0,\mathrm{free}}(\tau)-m_{\mathrm{pinned}}(\tau)\theta^s_{K,0,\mathrm{pinned}}(\tau)\right]^2\\+\left[m_{\mathrm{free}}(\tau)\eta^s_{K,0,\mathrm{free}}(\tau)-m_{\mathrm{pinned}}(\tau)\eta^s_{K,0,\mathrm{pinned}}(\tau)\right]^2\end{array}\right\}$$
$$\approx\left[r^{\mathrm{tot}}_{ss,0}(\tau)\right]^2[\sin^2(\alpha)+\sin^2(\alpha-2\theta)] \qquad (S25)$$

$$\frac{I^t_{\mathrm{P}+}(\tau)+I^t_{\mathrm{P}-}(\tau)}{I^i(\tau)}=\left[r^{\mathrm{tot}}_{ss,0}(\tau)\right]^2[\sin^2(\alpha)+\sin^2(\alpha-2\theta)]$$
$$+\left[r^{\mathrm{tot}}_{ss,0}(\tau)\right]^2[\cos^2(\alpha)+\cos^2(\alpha-2\theta)]\cdot\left\{\begin{array}{c}\left[m_{\mathrm{free}}(\tau)\theta^s_{K,0,\mathrm{free}}(\tau)+m_{\mathrm{pinned}}(\tau)\theta^s_{K,0,\mathrm{pinned}}(\tau)\right]^2\\+\left[m_{\mathrm{free}}(\tau)\eta^s_{K,0,\mathrm{free}}(\tau)+m_{\mathrm{pinned}}(\tau)\eta^s_{K,0,\mathrm{pinned}}(\tau)\right]^2\end{array}\right\} \qquad (S26)$$

These sums show no variation with time for AP (within the experimental error) and only a small drop of about 2% for P that can be attributed to the reductions of m_{free} and m_{pinned}. The approximation in equation (S25) comes from the fact that, in our experiments, magnetic signal in the AP configuration is smaller than in the P configuration. This expected because of the similarity of both probed TM sublattices. Thus, we conclude that transient reflectivity changes are smaller than our detection limit. Having shown that neither the magneto-optical nor the non-magnetic optical properties respond to the femtosecond laser excitation in any other way than via magnetization, we have proven that our analysis provides information about the true dynamics of m_{free} and m_{pinned}. Error bars are given by the standard deviation of the average of data obtained for all pairs of QWP angles i.e. the average of all the curves such as shown in Supplementary Fig. 9b.

TR-MOKE microscopy data analysis

The raw images are scaled along the horizontal axis to consider the probe beam angle of incidence. They are then modified and analyzed by our own image processing code, using the open source software ImageJ, that i) removes the noise related to random variations of probe laser intensity by equalizing the image brightness, ii) removes artifacts caused by dust on the sample or the optical imaging system by subtracting a background

image (average of five images taken at negative delay) from all images, iii) corrects for spatial variations of the pump laser spot by identifying the center of gravity of pump induced magnetization changes for each image and shifting the images to keep this center constant, iv) provides the response of the normalized magnetization in the P and AP configuration by subtracting the corrected images of pump induced magnetization changes, obtained after steps i)-iii) for opposite magnetization configurations at the same pump-probe delay, $P^+(\tau)$ - $P^-(\tau)$ and $AP^+(\tau)$ - $AP^-(\tau)$, adding the differences of the average intensities of the corresponding background images $<\Delta P>$ and $<\Delta AP>$, and dividing by $<\Delta P>$. Details of the image processing steps are as follows.

i) Equalization of image brightness. For each image, the program calculates the average intensity within a predefined area A that is not affected by the pump beam. These averages, $<I_{cal;i}(\tau)>_A$, calculated for images measured in all four experimental configurations i in $\{P^+, P^-, AP^+, AP^-\}$, are then plotted versus pump-probe delay τ and examined for systematic drifts. Only if the values show no systematic drift as those shown in Supplementary Fig. 9a, their averages over all pump-probe delays are defined as target for this configuration, $T_i = <<I_{cal;i}(\tau)>_A>_\tau$. The brightness of individual images is then equalized by multiplying their intensity by $T_i/<I_{cal;i}(\tau)>_A$. Measurements that resulted in systematic drifts of $<I_{cal;i}(\tau)>_A$ were discarded.

ii) Background subtraction. The average of five images taken at negative pump-probe delay for a certain magnetic configuration is calculated and defined as background image for this configuration. This background is subtracted from all images taken in this configuration, thereby removing artifacts caused by dust or defects on the sample or the optical imaging system. A typical image taken in the P^+ configuration at τ = 1 ps is shown before and after background subtraction in Supplementary Fig. 6b and 6c respectively. Note, that the intensity of the background corrected images is now a measure of pump induced changes of the intensity in the raw image. Thus these images are analogous to Kerr signals measured in non-imaging set-ups with pump chopped lock-in detection. The probe angle of incidence induces an horizontal scaling of the images which is corrected based on the measured probe angle of incidence.

iii) Correction for variations of pump spot position. Duplicates of all images are converted to binary with the criterion that all pixels with their absolute value within a certain upper percentile (typically 95 %) are set to one and all others to zero. The coordinates of the center of gravity (CG) of these binary images is calculated and stored. The CG of the image measured in the P^+ configuration at the longest delay is defined as target, CG_0, and all raw images are shifted to bring the CG of their binary counterpart into overlap with CG_0. However, images taken at short pump-probe delay (typically τ < 1.5 ps) are all shifted by the same amount as the image measured for τ = 1.5 ps. This ensures that the real, systematic movement/deformation of the measured profile of pump induced magnetization changes due to the variation of the pump-probe delay with horizontal position x (the coordinate along the line of intersection between the sample and the plane spanned by the non-collinear pump and probe beams) is not artificially removed (further information about the interdependence of x and delay is given in the Supplementary Information). Finally, the images are cropped to the intersecting area of all images.

iv) Determination of magnetization dynamics for different pump fluences.

The detailed description about how the magnetization dynamics of each layer is retrieved is provided above. Zero pump-probe delay for short time step measurements is defined when the largest magnetization change happens, which is suitable for our relatively large probe pulse duration. The dynamics for different fluence is obtained by considering different vertical positions on the microscopy images for a given horizontal position. It was verified that identical results are obtained by considering different horizontal positions. For each position, the dynamics is averaged over a square of 10 µm size. The fluence is obtained from the measured laser power and the gaussian profile of the beam. The uncertainty on the fluence is mostly determined by this averaging procedure and not by the small fluctuations of the beam position during image acquisition. The uncertainty then depends on the position on the image. At the maximum fluence of 5.08 mJ/cm^2, the uncertainty is ± 0.01 mJ/cm^2 while it reaches ±0.28 mJ/cm^2 for 3.03 mJ/cm^2. We note that this relatively larger uncertainty cannot solely explain the observed CSD around the threshold fluence (due to a

possible averaging of magnetization dynamics where the magnetization reverses at higher fluences and recovers for lower ones).

Details about the numerical resolution of the model.

This system of equation (2TM plus magnetization dynamics) in one spatial dimension is solved by discretizing the sample and using the boundary conditions detailed in reference[6] with all interfaces transparent to heat transport except for the metal/substrate interface where an interfacial thermal conductance G was assumed to exist (for the phonon temperature). To model the boundary condition for the phonon temperature at an infinite depth in the substrate, we model a thin section of substrate (10 nm) and ask that $\left(T_p(z+\Delta z)-RT\right)/\left(T_p(z)-RT\right)=\left(T_p(z)-RT\right)/\left(T_p(z-\Delta z)-RT\right)$ at the largest simulated depth $z+\Delta z$, for a spatial step Δz. This is the condition satisfied by gaussian and exponential solutions. For the simulated timescales in this work, the exact boundary condition that we use does not have a visible impact. Because of the non-linearity of our 2TM, the numerical scheme used to solve it is a simple forward Euler method. Spatial and time steps are always checked to ensure convergence of the solution. In this work, we used a spatial step $\Delta z=0.25$ nm and a time step $\Delta \mathrm{t}=0.01$ fs, the latter increment also allowing us to fully consider that fast oscillations in the absorption calculated as explained above. We note that for the studied systems, our method to calculate absorption is relatively close to what is obtained using the standard TMM but this is not the case in general.

The parameters used for the 2TM are shown in Supplementary Table 1. The interfacial heat conductance at the interface with the substrate we used was $G=100\ 10^{6}$ $\mathrm{Wm^{-2}K^{-1}}$ taken from reference[6] but it was found unimportant for the timescales involved in these simulations. We used the same parameters as in our experimental setup for optical calculations. The only parameters required from the literature are the complex and frequency dependent optical indices. The reference for each material is shown Supplementary Table 1. For the magnetization dynamics simulations of the [Co/Pt] multilayer, we used $T_c=500$ K, $\tau=0.132$ ps as obtained using the microscopic Three Temperature Model[7] with the parameters from reference[8], $\rho=1$ $\mathrm{eV^{-1}}$

as in reference[9] and $\tau_s = 35$ fs. The value of τ_s is one of the most important parameter of these simulations as it gives the amplitude of the spin accumulation. The small value that we used is reasonable considering typical spin relaxation times in ferrimagnets[10] and ferromagnets[11] below 0.1 ps. Values of τ_s with an order of magnitude smaller would be unrealistic. Higher values increase the magnetization reversal speed and reduce its required threshold fluence. The value of the parameter ρ does not influence the energy efficiency or speed of the reversal in this model but only affect the (transient) contribution of the spin accumulation to the magnetization whose value is much smaller than the equilibrium magnetization of the ferromagnetic multilayer. Decreasing T_c results in a smaller reversed amplitude (thus a better match with the experiments on a long timescale) but (i) reduces the threshold fluence and (ii) makes the three step dynamics (ultrafast demagnetization; non equilibrium recovery; equilibrium recovery) less obvious (the dynamics looks like what we obtained at higher fluence in Supplementary Fig. 11c). In order to match the magnetization of GdFeCo at room temperature for our alloy composition[12], we used a number of atoms per unit volume of 2.5 10^{28} m^{-3} to convert the magnetic moment obtained from reference[13] into magnetization.

Supplementary Note

Spin temperature in the microcanonical ensemble

In order to define the temperature of an ensemble of localized spins, independent of any additional bath, we consider a system described by the following Hamiltonian in the microcanonical ensemble:

$$H = -\frac{\delta}{N}\sum_{i\neq j} S_i^z \langle S_j^z \rangle \qquad (S27)$$

Where S_i^z are spin operators in the direction of the quantization axis z, N is the total number of spins in the system and angular brackets denote average for the density matrix ρ of the system in the microcanonical ensemble. The constant δ is related to the Curie temperature of the system below. It can represent different

physical quantities depending on the microscopic origin of the exchange interaction. This Hamiltonian is the usual mean field Hamiltonian[14] where we have not used the fact that $\langle S_j^z \rangle$ is independent of the spin site j. This will be important to obtain the correct expression for the spin entropy and hence the spin temperature. Defining the normalized magnetization $m = -\langle S_i^z \rangle / S$, independent of spin site, where S is the spin quantum number, and focusing on the two level system case ($S = 1/2$), the energy of the system depends on magnetization via $E = \langle H \rangle = -\delta N m^2/4$. We consider as in Figure 1 that the lowest energy level corresponds to the -1/2 component of S_i^z such that $m = 1$ when this level is fully occupied. Our final result is independent of this choice. To calculate the entropy at a given system energy, one needs to know the trace of the density matrix for that energy[15]. A diagonal density matrix element ρ_k will be equal to 1 (the final result does not depend on the choice of this value) if the state $|k\rangle$ represents the system in a configuration such that its energy is E, and 0 otherwise. The number of such states $\mathrm{Tr}(\rho) = \sum_k \rho_k$ may a priori be evaluated to be $\binom{N}{n}$ where $n = N(1 - m(E))/2$ is the number of spins in the excited state ($\langle k|S_i^z|k\rangle = 1/2$) as in the case of a paramagnet. However, as the double sum in equation (S27) shows, one must consider that each spin feels a mean field, generated by the other spins. All the possible spin configurations leading to an identical mean field must be accounted for in the evaluation of the entropy. Such mean fields $\langle S_j^z \rangle$, although having the same value, are uncorrelated ($\langle \langle S_j^z \rangle \langle S_i^z \rangle \rangle = \langle S_i^z \rangle \langle S_j^z \rangle$). Thus for each of the $\binom{N}{n}$ spin configurations, there are $\binom{N-1}{n}$ configurations of the mean field which correspond to a system with (almost) the same energy E. Note that we consider macroscopic systems at finite temperature where both N and n are large numbers. In this limit, the entropy is then given by:

$$S(E) = k_B \ln\big(\mathrm{Tr}(\rho)\big) \approx 2k_B \ln \binom{N}{n} \qquad (S28)$$

Using Stirling's approximation and the fact that $m = 1 - 2n/N$, one gets:

$$S(E) = 2k_B \left[N \ln(2) - \frac{N}{2} \ln(1 - m^2) - Nm \tanh^{-1}(m) \right] \qquad (S29)$$

Finally, the spin temperature is obtained from:

$$T_S = \frac{1}{\left(\frac{\partial S}{\partial E}\right)} = \frac{\delta m}{4k_B \tanh^{-1}(m)} \qquad (S30)$$

This can be rewritten:

$$m = \tanh\left(\frac{\delta m}{4k_B T_S}\right) = \tanh\left(\frac{mT_C}{T_S}\right) \qquad (S31)$$

Which is the usual self consistent mean field equation for magnetization, as usually obtained in the canonical ensemble, if T_S is replaced by the temperature of an external reservoir. The Curie temperature T_C is then defined, as usual, as the spin temperature above which equation (S31) only has the solution $m = 0$. This allows us to have the usual identification $\delta = 4k_B T_C = 3k_B T_C/(S(S+1))$[14] (written as $\Delta = \delta(-\langle S^z\rangle) = 2k_B T_c(-\langle S^z\rangle/S) = 2mK_B T_C$ by Beens et al.[9]) leading to equation (1) in the main text. We note that if the entropy of the mean field $S_{\text{mean field}} = k_B \ln\binom{N-1}{n}$ is not considered, as one might be tempted to do if the Hamiltonian is written $H = -\delta\langle S^z\rangle \sum_i S_i^z$, the obtained identification would be $\delta = 2k_B T_C$.

This construction of temperature usually relies on the axioms of quantum statistical mechanics[15]: the postulate of equal a priori probability and the postulate of random phases. This permits to have a diagonal density matrix whose elements are all equal to the same constant (chosen here to be 1) or zero. We briefly discuss why it is still a reasonable approximation to consider this form of density matrix in our case for the localized spin system. The density matrix is diagonal in equilibrium by definition (this condition is also achieved in our experiments) and it remains this way after interaction with a laser pulse since the magnetization dynamics is longitudinal. Would the density matrix not be diagonal, one could still define entropy as discussed for instance by Snoke et al.[16]. It is not so clear however that we can safely consider that the density matrix elements are all equal to the same constant or zero. Indeed, it could be possible that certain spin configurations, leading to a given magnetization, are systematically favored compared to others (even for different pulses in our TR-MOKE experiments) due to the fact that the laser pulse will lead to specific electronic excitations for a given material and wavelength. However, since ultrafast demagnetization is

strongly independent on the nature of those excitations[8,17,18] and because many interactions between spins and electrons are expected to happen during a time window corresponding to the time resolution of our experiment (~200fs), as it is comparable with the characteristic ultrafast demagnetization time, we believe that the postulate of equal a priori probabilities is fulfilled in the magnetization dynamics that we observe.

This way, we can define a temperature without the notion of equilibrium which allows us to make a link between a disorder (and hence temperature) and possible sets of spin configurations (and hence magnetization) at any given instant. We note that in equilibrium and for an external bath temperature T below T_C, we have $T = T_S$ while both temperatures will be different out of equilibrium. This justifies the denomination of three temperature models for models where one equation is actually an equation for m rather than T_S[7,9]. An important difference between the canonical and microcanonical ensembles description of a system of localized spins is that in the microcanonical sense, the spin temperature cannot be higher than the Curie temperature while in the canonical sense, temperature can be as high as desired because it is the temperature of an external bath.

Finally, we note that the spin temperature can be generalized to any number of levels (i.e. spin quantum number) following the same procedure.

Analysis of equation (12) regarding ultrafast magnetization reversal

Equation (12) has several properties worth mentioning especially when the spin accumulation is different from 0. This equation describes the transfer of angular momentum between the localized and itinerant electrons which are assumed to be two well distinct systems[9]. The speed of the transfer, i.e. $\mathrm{d}m/\mathrm{d}t$, depends on the characteristic time τ but also on the localized normalized magnetic moment m and the spin accumulation $\Delta\mu$. In the absence of spin accumulation, when m approaches 0, $\left[1 - m\coth\left(\frac{2mk_BT_C - \Delta\mu}{2k_BT_e}\right)\right]$ is finite (possibly even 0) and $\mathrm{d}m/\mathrm{d}t$ also approaches 0 such that the overall transfer of angular momentum vanishes, which is a manifestation of CSD of magnetization dynamics, a feature appearing in several other models of ultrafast magnetization dynamics[7,19-22]. The situation is drastically different when $\Delta\mu \neq 0$. In this

case, when m approaches 0, $\left[1 - m\coth\left(\frac{2mk_BT_C-\Delta\mu}{2k_BT_e}\right)\right]$ is still finite but $\mathrm{d}m/\mathrm{d}t \approx -\Delta\mu/(2\tau k_BT_C) \neq 0$ i.e. the higher the spin accumulation, the faster the magnetization reversal occurs meaning that the spin accumulation prevents any critical slowing down from happening. It is worth noting that when $m - \frac{\Delta\mu}{2k_BT_C} \approx 0$, corresponding to $\Delta E + \Delta E_{\text{ext}} = 0$, $\left[1 - m\coth\left(\frac{2mk_BT_C-\Delta\mu}{2k_BT_e}\right)\right]$ is infinite such that $\mathrm{d}m/\mathrm{d}t \approx -mT_e/(\tau T_C)$ is also finite. The conclusion is that in all cases, as long as the spin accumulation is not zero, equation (12) does not yield $\mathrm{d}m/\mathrm{d}t = 0$ when m approaches 0. Equation (12) may be written $\mathrm{d}m/\mathrm{d}t = -\left(m(t) - m_{eq}^*(t)\right)/\tau^*(t)$ where $m_{eq}^*(t) = \mathrm{th}\left(\frac{2m(t)k_BT_C-\Delta\mu(t)}{2k_BT_e(t)}\right)$ and $\tau^*(t) = m_{eq}^*(t)\tau/\left(m(t) - \frac{\Delta\mu(t)}{2k_BT_C}\right)$, showing that m tends towards m_{eq}^* with a characteristic time that depends on the current value of m (causing critical slowdown when m approaches 0 in the absence of spin accumulation even if $m_{eq}^* \neq 0$), electronic temperature and spin accumulation. Defining $m_{eq}(t)$ as the solution of the self consistent equation $m_{eq} = \mathrm{th}\left(\frac{2m_{eq}k_BT_C-\Delta\mu(t)}{2k_BT_e(t)}\right)$, equilibrium between localized and itinerant electrons is reached when $\mathrm{d}m/\mathrm{d}t = 0$ permanently which is equivalent to $m = m_{eq}$ and $\mathrm{d}m/\mathrm{d}t = \mathrm{d}m_{eq}/\mathrm{d}t$, which is only rigorously verified after an infinite time. It is possible that $m = m_{eq}$ is satisfied at one point in time but not $\mathrm{d}m/\mathrm{d}t = \mathrm{d}m_{eq}/\mathrm{d}t$; this happens when $\mathrm{d}m/\mathrm{d}t$ changes sign. Both m and m_{eq} are plotted in Supplementary Fig. 11e for a specific fluence and a P configuration. The effect of the spin accumulation is greater when m is sufficiently small. Equation (12) shows that the magnetization dynamics starts being dominated by the spin accumulation when $2mk_BT_C \lesssim \Delta\mu$ i.e. $\Delta E \lesssim \Delta E_{\text{ext}}$. Thus, at small enough fluences (where the generated spin accumulation is also smaller), one expects to obtain the same magnetization dynamics whether there is a spin accumulation or not which is indeed what is observed in Figure 4 in the AP configuration. Finally, we note that the spin accumulation directly contributes to the total magnetization dynamics $m_{tot} = m - \rho\,\Delta\mu$. With our value of ρ, we find however that $\rho\,\Delta\mu$ cannot be higher than 0.014 at any instant and for any fluence in our simulations. This is consistent with the fact that the measured spin current induced magnetization in normal metals is orders of magnitude smaller than the magnetization inside ferromagnets[10].

Influence of laser beam angles of incidence in TR-MOKE microscopy measurements

Because of the angles of incidence that we have in our TR-MOKE microscope, the CG of the signal moves as a function of time at short pump-probe delays. This is clearly visible on the experimental data shown in Supplementary Fig. 7. Some signal starts being visible on the left of the microscope image (mirrored compared to what is really happening on the sample) at a delay of around -500 fs. Its CG then moves towards the center of the image and stabilizes in around 1 ps. Due to an angle of incidence, a laser pulse irradiates the sample at different instants depending on the considered position on the sample. Two positions separated by a distance Δx in the horizontal direction will be irradiated with a time delay $\Delta x \sin(\alpha)/c$ where α is the considered laser beam angle of incidence and c is the speed of light. The signal CG motion is due to different pump-probe delays for different horizontal positions on the sample. If the pump and probe beams had the same angle of incidence, even if it was not normal incidence, the observed signal CG would be fixed because the pump-probe delay would be the same everywhere. The pump-probe delay shift Δt between two positions separated by a distance Δx in the horizontal direction is then given by:

$$\Delta t = \frac{\Delta x \left(\sin(\alpha_{pump}) - \sin(\alpha_{probe})\right)}{c} \qquad (S32)$$

Where we consider oriented angles. Using this fact, we can reproduce the observed signal CG shift using the results of our simulations. We performed calculations for 100 uniformly distributed positions along the vertical direction (perpendicular to the plane of incidence), where no pump-probe delay shift is expected, from the center of the beam to a position 216 µm away from the center. The pump fluence at each position is given by the Gaussian profile for a beam with a diameter of 288 µm as in the experiments. The results are then extrapolated to all positions where we considered the pump-probe delay shift along the horizontal direction as well as the pump angle of incidence (resulting in a slightly elliptical signal even at long time delays). The results are shown in Supplementary Fig. 8 and closely resemble the experimental data (the pump beam is supposed to be circular and the beam diameter was chosen to be 288 µm). The color map is generated with the "pcolormesh" function of the Matplotlib library for Python. The shading parameter is 'gouraud' and

the color map parameter is 'bwr' with maximum and minimum value given by the equilibrium normalized magnetization at room temperature. We note that the pattern appearing in the simulations becomes sharp after a few picoseconds, contrary to the experiments, because, as discussed in the main body of this work, our simulations do not reproduce correctly the magnetization dynamics for fluences around the threshold fluence. We also remind the reader that the signal observed in Supplementary Fig. 7 corresponds to a mixture of Kerr rotation and ellipticity induced by two different magnetic layers while the simulation in Supplementary Fig. 8 show magnetization of a single magnetic layer. Thus we cannot reproduce the annular pattern of Supplementary Fig. 7b. The white area in Supplementary Fig. 8 appears because the sample temperature is significantly higher in the center of the illuminated area and thus the resulting equilibrium magnetization is lower.

Supplementary Table

Supplementary Table 1 | Parameters used for the 2TM

	γ ($JK^{-2}m^{-3}$)	κ_e ($WK^{-1}m^{-1}$)	g_{e-p} (10^{16} $WK^{-1}m^{-3}$)	C_p (10^6 $JK^{-1}m^{-3}$)	κ_p ($WK^{-1}m^{-1}$)	$n(\omega)$
[Co/Pt]	720[8]	20[8]	264[8]	2.98[8]	1[6]	[23]
Cu	98[24]	300[8]	7.5[8]	2.63[8]	5[6]	[25]
Pt	749[24]	45[8]	110[8]	3.45[8]	5 (same as Cu)	[26]
Ta	543[24]	58[24]	100[24]	2.23[24]	5 (same as Cu)	[26]
GdFeCo	781[24]	10.5[24]	60[24]	2.3[24]	5 (same as Cu)	[27]
Sapphire	0	0	0	3.16	30[6]	[28]
Glass	0	0	0	2	2	1.5136

Supplementary Information References

1. Hamrle, J. Magneto-optical determination of the in-depth magnetization profile in magnetic multilayers. Ph.D. thesis (Université Paris-Sud XI, 2003).
2. Sato, K. & Togami, Y. Magneto-optical spectra of RF-sputtered amorphous Gd-Co and Gd-Fe films. *J. Magn. Magn. Mater.* **35**, 181–182 (1983).
3. Guidoni, L., Beaurepaire, E. & Bigot, J.-Y. Magneto-optics in the Ultrafast Regime: Thermalization of Spin Populations in Ferromagnetic Films. *Phys. Rev. Lett.* **89**, 017401 (2002).

4. Koopmans, B., Kampen, M. van & Jonge, W. J. M. de. Experimental access to femtosecond spin dynamics. *J. Phys. Condens. Matter* **15**, S723–S736 (2003).
5. Razdolski, I. *et al.* Analysis of the time-resolved magneto-optical Kerr effect for ultrafast magnetization dynamics in ferromagnetic thin films. *J. Phys. Condens. Matter* **29**, 174002 (2017).
6. Kimling, J. & Cahill, D. G. Spin diffusion induced by pulsed-laser heating and the role of spin heat accumulation. *Phys. Rev. B* **95**, 014402 (2017).
7. Koopmans, B. *et al.* Explaining the paradoxical diversity of ultrafast laser-induced demagnetization. *Nat. Mater.* **9**, 259–265 (2010).
8. Bergeard, N. *et al.* Hot-Electron-Induced Ultrafast Demagnetization in Co / Pt Multilayers. *Phys. Rev. Lett.* **117**, 147203 (2016).
9. Beens, M., Duine, R. A. & Koopmans, B. S-d model for local and nonlocal spin dynamics in laser-excited magnetic heterostructures. *Phys. Rev. B* **102**, 054442 (2020).
10. Choi, G.-M. & Min, B.-C. Laser-driven spin generation in the conduction bands of ferrimagnetic metals. *Phys. Rev. B* **97**, 014410 (2018).
11. Lichtenberg, T., Beens, M., Jansen, M. H., Koopmans, B., & Duine, R. A. Probing optically induced spin currents using terahertz spin waves in noncollinear magnetic bilayers. *Phys. Rev. B* **105**, 144416 (2022).
12. Ding, M. & Poon, S. J. Tunable perpendicular magnetic anisotropy in GdFeCo amorphous films. *J. Magn. Magn. Mater.* **339**, 51–55 (2013).
13. Radu, I. *et al.* Transient ferromagnetic-like state mediating ultrafast reversal of antiferromagnetically coupled spins. *Nature* **472**, 205–208 (2011).
14. Nolting, W. & Ramakanth, A. *Quantum Theory of Magnetism*. (Springer Berlin Heidelberg, 2009).
15. Huang, K. *Statistical Mechanics, 2nd Edition*. *Statistical Mechanics* (John Wiley & Sons, 1987).
16. Snoke, D. W., Liu, G. & Girvin, S. M. The basis of the Second Law of thermodynamics in quantum field theory. *Ann. Phys. (N. Y).* **327**, 1825–1851 (2012).
17. Chekhov, A. L. *et al.* Ultrafast Demagnetization of Iron Induced by Optical versus Terahertz Pulses. *Phys. Rev. X* **11**, 041055 (2021).
18. Pellegrini, C., Sharma, S., Dewhurst, J. K. & Sanna, A. Ab initio study of ultrafast demagnetization of elementary ferromagnets by terahertz versus optical pulses. *Phys. Rev. B* **105**, 134425 (2022).
19. Kazantseva, N., Nowak, U., Chantrell, R. W., Hohlfeld, J. & Rebei, A. Slow recovery of the magnetisation after a sub-picosecond heat pulse. *EPL (Europhysics Lett.* **81**, 27004 (2008).
20. Atxitia, U., Chubykalo-Fesenko, O., Walowski, J., Mann, A. & Münzenberg, M. Evidence for thermal mechanisms in laser-induced femtosecond spin dynamics. *Phys. Rev. B* **81**, 174401 (2010).
21. Manchon, A., Li, Q., Xu, L. & Zhang, S. Theory of laser-induced demagnetization at high temperatures. *Phys. Rev. B* **85**, 064408 (2012).
22. Xu, L. & Zhang, S. Magnetization dynamics at elevated temperatures. *Phys. E Low-dimensional Syst. Nanostructures* **45**, 72–76 (2012).
23. Atkinson, R. *et al.* Fundamental optical and magneto-optical constants of Co/Pt and CoNi/Pt multilayered films. *J. Magn. Magn. Mater.* **162**, 131–138 (1996).

24. Igarashi, J. *et al.* Engineering Single-Shot All-Optical Switching of Ferromagnetic Materials. *Nano Lett.* **20**, 8654–8660 (2020).

25. McPeak, K. M. *et al.* Plasmonic Films Can Easily Be Better: Rules and Recipes. *ACS Photonics* **2**, 326–333 (2015).

26. Werner, W. S. M., Glantschnig, K. & Ambrosch-Draxl, C. Optical Constants and Inelastic Electron-Scattering Data for 17 Elemental Metals. *J. Phys. Chem. Ref. Data* **38**, 1013–1092 (2009).

27. Hendren, W. R. *et al.* Optical and magneto-optical characterization of TbFeCo and GdFeCo thin films for high-density recording. *J. Phys. Condens. Matter* **15**, 1461–1468 (2003).

28. Malitson, I. & Dodge, M. Refractive-index and birefringence of synthetic sapphire. *J. Opt. Soc. Am.* **62**, 1405 (1972).
